# Follistatin Mitigates Atherosclerosis Through Activation of Arginine Metabolism and Adipose Browning

**DOI:** 10.3390/cells15131205

**Published:** 2026-07-02

**Authors:** Golnaz Dirakvand, Shehla Pervin, Brian Villa, Christy Le, Kristine Yohanna, Victor Grijalva, Arnab Chattopadhyay, Satyesh K. Sinha, Srinivasa T. Reddy, Rajan Singh

**Affiliations:** 1 Department of Biology, California State University, Dominguez Hills, Los Angeles, CA 90747, USA; 24g.dirakvand@gmail.com (G.D.); shehlapervin@cdrewu.edu (S.P.); bvilla2018@gmail.com (B.V.); christyyyl3@gmail.com (C.L.); kristineyohanna@cdrewu.edu (K.Y.); 2 Division of Endocrinology and Metabolism, College of Medicine, Charles R. Drew University of Medicine and Science, Los Angeles, CA 90747, USA; 3 Department of Obstetrics and Gynecology, David Geffen School of Medicine, University of California Los Angeles, Los Angeles, CA 90747, USA; 4 Division of Cardiology, Department of Medicine, David Geffen School of Medicine, University of California Los Angeles, Los Angeles, CA 90747, USA; vgrijalva@mednet.ucla.edu (V.G.); arnabucla@gmail.com (A.C.); sksinha@mednet.ucla.edu (S.K.S.); sreddy@mednet.ucla.edu (S.T.R.)

**Keywords:** follistatin, low-density lipoprotein, adeno-associated virus, arginase 1, fibroblast growth factor 21, mouse embryonic fibroblast, white adipose tissue

## Abstract

Follistatin (FST) binds to and neutralizes members of the transforming growth factor-beta (TGF-β) superfamily, thereby regulating diverse physiological processes, including regulation of skeletal muscle, adipose, and bone homeostasis. FST also promotes adipose browning and enhances energy metabolism, leading to improved plasma lipid profiles and metabolic health in mice. Given the emerging association between brown adipose tissue (BAT) activation and reduced atherosclerosis, we investigated the anti-atherogenic potential of FST. Transcriptomic and metabolomic analyses of the Hybrid Mouse Diversity Panel (HMDP) revealed that *Fst* expression was negatively correlated with aortic lesion area and positively correlated with the expression of multiple adipose browning-associated genes. Adeno-associated viral delivery of Fst (AAV1-FST344) in *Ldlr*^−/−^ mice significantly reduced aortic lesion area, improved plasma lipid profiles, and decreased expression of adhesion (VCAM1) and inflammatory (iNOS, TNF-α) markers in white adipose tissue (WAT), liver, and heart. *Fst* gene delivery also markedly increased uncoupling protein 1 (UCP1) expression in WAT, consistent with WAT browning. Integrated correlation analyses of *Fst* expression with tissue metabolites, together with plasma metabolite–lesion associations identified in the HMDP, implicated the arginase 1 (Arg1)-mediated metabolic pathway as a key regulator of atherogenesis. Consistent with these findings, Arg1 expression was significantly elevated in WAT, liver, and heart of AAV1-FST344-treated mice and in wild-type versus *Fst*-knockout mouse embryonic fibroblasts (MEFs). Immunostaining localized Arg1 predominantly to CD68^+^ macrophages in heart and liver. Given recent evidence identifying Arg1 as a novel mediator of efferocytosis, these findings suggest that Arg1 may promote macrophage metabolic reprogramming and resolution of inflammation by enhancing the clearance of apoptotic cells. Furthermore, *Fst* gene delivery increased the expression of fibroblast growth factor 21 (*Fgf21*) and adiponectin (AdipoQ) in WAT. Collectively, these findings identify *Fst* as a novel anti-atherogenic regulator that protects against vascular disease by promoting adipose browning, improving lipid metabolism, and activating Arg1-mediated metabolic pathways.

## 1. Introduction

Follistatin (FST) is a secreted glycoprotein that regulates multiple physiological processes by binding to and neutralizing TGF-β superfamily, particularly activins, myostatin, and certain bone morphogenic proteins (BMPs). FST exhibits exceptionally high affinity for Activin-A (picomolar to sub-nanomolar range), allowing it to outcompete activin receptors (ActRIIA/ActRIIB), and effectively suppress downstream SMAD2/3 signaling, while binding myostatin and selected BMPs with relatively lower affinity. Through its circulating (FST315) and tissue-bound (FST288) isoforms, FST modulates muscle growth, metabolism, inflammation, fibrosis, and tissue repair, making it an important therapeutic target for metabolic, cardiovascular, muscular, and fibrotic diseases [1,2,3]. Initially identified as a regulator of follicle-stimulating hormone (FSH) secretion and skeletal muscle growth, FST has recently emerged as an important modulator of lipid and energy metabolism [4,5]. In adipose tissue, FST promotes the transformation of white adipose tissue (WAT) into brown adipose tissue (BAT)-like phenotype by activating multiple thermogenic signaling pathways [4,5]. This phenotypic conversion increases energy expenditure, improves insulin sensitivity, and alleviates obesity-associated cardiovascular complications [4,5,6]. Our previous studies demonstrated that FST activates UCP1, AMP-activated protein kinase (AMPK), and peroxisome proliferator-activated receptor gamma coactivator 1-alpha (PGC1α), key regulators of mitochondrial biogenesis, thereby improving plasma lipid profiles and metabolic function in experimental mice [4,5]. FST is expressed in multiple tissues, including skeletal muscle, liver, bone, and adipose tissue, with expression levels varying according to developmental stage and physiological context [4].

Atherosclerosis, the underlying cause of most cardiovascular diseases (CVD), is a multifactorial disorder driven by hyperlipidemia, inflammation, and metabolic dysfunction [7,8]. Collectively, CVDs are projected to account for approximately 23.3 million deaths worldwide by 2033 and impose an estimated economic burden of $368 billion in the United States by 2035 [9]. Atherosclerosis is characterized by lipid accumulation and chronic inflammation within the arterial wall, resulting in plaque formation and progressive vascular dysfunction [7,8]. Emerging evidence suggests that FST-induced adipose browning may provide cardiovascular protection, as activation of BAT has been associated with attenuation of atherosclerosis [10,11,12]. Both preclinical and clinical studies have demonstrated an inverse relationship between WAT browning and atherosclerotic plaque burden [10,13,14]. ^18^F-FDG PET/CT imaging in 443 volunteers demonstrated an inverse correlation between BAT activity, arterial inflammation, and cardiovascular events [15]. Similarly, treatment with the β3-adrenergic receptor agonist BRL37344 significantly reduced triglycerides, total cholesterol, improved insulin sensitivity and dose-dependently reduced atherosclerotic lesion formation in *apoE*^−/−^ mice fed a high-fat diet [16]. Interestingly, several TGF-β superfamily members, including Activin-A and myostatin (MST), are expressed within atherosclerotic lesions, and genetic disruption of *Mst* attenuates plaque development [17,18]. However, the direct role of FST in atherosclerosis remains largely unknown.

To investigate this question, we analyzed transcriptomic and metabolomic datasets from the Hybrid Mouse Diversity Panel (HMDP), a systems genetics resource comprising more than 100 inbred strains that enables high-resolution mapping of molecular and metabolic traits [19,20]. These analyses revealed significant associations between aortic *Fst* expression and atherosclerosis-related phenotypes, as well as key metabolic intermediates. Integration of gene expression, metabolomic, and lesion datasets further identified potential links between *Fst* expression and the arginine-ornithine metabolic pathway, a critical regulator of vascular inflammation and endothelial function. Given prior evidence that adipose tissue browning, lipid metabolism, and arginine-urea cycle enzymes contribute to protection against metabolic and vascular disease, we hypothesized that FST attenuates atherosclerosis by coordinated regulation of metabolic and inflammatory pathways. To test this hypothesis experimentally, we delivered the biologically active spliced form of *Fst* (AAV1-FST344) via adeno-associated viral vector (AAV1) in *Ldlr*^−/−^ mice fed a western diet (WD). FST-treated mice exhibited significantly improved plasma lipid profiles, reduced aortic lesion area, and decreased expression of inflammatory (iNOS) and adhesion (VCAM1) markers in liver, WAT, and heart compared with control groups. *Fst* gene delivery also induced UCP1 expression in WAT, consistent with enhanced adipose browning. Importantly, *Fst* markedly upregulated Arg1 expression in the liver, WAT, and heart, accompanied by increased tissue expression and circulating levels of *Fgf21* and AdipoQ.

Collectively, these findings identify *Fst* as a novel regulator of anti-atherogenic metabolic pathways. By promoting adipose browning, improving lipid metabolism, and upregulating Arg1 and *Fgf21*-mediated signaling, *Fst* protects against dyslipidemia and attenuates the development of atherosclerosis in mice.

## 2. Materials and Methods

### 2.1. Hybrid Mouse Diversity Panel (HMDP)

The Hybrid Mouse Diversity Panel (HMDP) is a systems genetics resource consisting of approximately 100 genetically diverse inbred mouse strains, developed by a multidisciplinary consortium of investigators from multiple institutions [19]. HMDP integrates genomic, transcriptomic, metabolomic, proteomic, and phenotypic datasets generated under standardized experimental conditions and has been extensively validated for the study of complex traits, including cardiovascular and metabolic diseases.

### 2.2. Animals

All animal procedures were approved by the Institutional Animal Care and Use Committee (IACUC). Studies involving *Ldlr*^−/−^ mice were approved by the Institutional Animal Care and Use Committee of the Lundquist Institute (Protocol I911-161; 22457-02). Bone marrow was isolated from 6- to 12-week-old C57BL/6 mice under protocols approved by the UCLA IACUC (Protocol ARC-2010-003).

Eight- to ten-week-old male low-density lipoprotein receptor-deficient (*Ldlr*^−/−^) mice received tail-vein injections of 5 × 10^11^ viral particles of either AAV1-null (control) or AAV1-FST344 (Vector BioLabs, Malvern, PA, USA). Following viral administration, mice were maintained on a WD for 12 weeks. Liver, WAT, and heart tissues were harvested for gene and protein expression analyses by Western blotting, immunohistochemistry, and quantitative real-time PCR. Male and female *Fst* heterozygous (*Fst*^+/−^) mice were obtained from Dr. Martin Matzuk (Baylor College of Medicine, Houston, TX, USA) [3]. Breeding pairs of *Fst*^+/−^ mice were mated, and embryos were collected at embryonic day 13.5 (E13.5). Embryos were genotyped to establish primary mouse embryonic fibroblast (MEF) primary cultures from wild-type (WT) and *Fst* knockout, (KO) littermates, as described previously [4,5].

All mice were housed under controlled environmental conditions (23 °C; 12 h light/12 h dark cycle) with free access to food and water. To minimize experimental bias, age- and sex-matched *Ldlr*^−/−^ mice were randomly assigned to receive either AAV1-null or AAV1-FST344. Investigators remained blinded during lesion quantification and histological analyses to evaluate the effect of follistatin on atherosclerosis development.

### 2.3. MEF Culture

MEFs were isolated from E13.5 embryos as previously described [4,5]. Briefly, embryos were harvested, and the head, limbs, and internal organs were removed. The remaining carcasses were rinsed with 1× phosphate-buffered saline (PBS), minced into small fragments, and digested with 3 mL of 0.025% trypsin–EDTA (Invitrogen, Carlsbad, CA, USA) at 37 °C for 20 min using two successive trypsinization cycles. Trypsin activity was neutralized by adding an equal volume of culture medium (Dulbecco’s modified Eagle’s medium [DMEM] supplemented with 10% fetal bovine serum [FBS], 20 mM L-glutamine, and penicillin/streptomycin). Cell suspensions were centrifuged, resuspended in fresh medium, and plated in T-75 culture flasks. MEFs were maintained at 37 °C in a humidified incubator containing 5% CO_2_ and 95% air.

### 2.4. Genotyping

Genomic DNA was isolated from the heads of each harvested embryo using Direct Lysis Reagent (Viagen Biotech, Los Angeles, CA, USA). Genotypes were determined by PCR using allele-specific primers. The following primer sets were used: Wild-type (WT) *Fst* allele: 5′-ATCTATCGCCCTTGGGTCTT-3′ (forward) and 5′-AAAACCTACCGCAACGAATG-3′ (reverse), producing a 152 bp amplicon. Knockout (KO) *Fst* allele: 5′-GGTGGGAAATGTCACCTGAT-3′ (forward) and 5′-CGGTGGATGTGGAATGTGT-3′ (reverse), producing a 262 bp amplicon.

PCR amplification was performed under the following conditions: initial denaturation at 94 °C for 1 min, followed by 15 cycles of 94 °C for 30 s and 68 °C for 30 s (with a temperature decrease of 0.5 °C per cycle); followed by 20 cycles of 94 °C for 30 s and 60 °C for 30 s. PCR products were resolved on 2% agarose gels and visualized under UV illumination [4,5].

### 2.5. Plasma Lipid Analysis

Serum triglycerides (TG), free fatty acids (FFA), total cholesterol (TC), and high-density lipoprotein cholesterol (HDL-C) levels were measured using enzymatic colorimetric assays, as described previously [21,22].

### 2.6. ELISA

Serum concentrations of *Fst* (Cat# DFN00), *Fgf21* (Cat# MF2100), and AdipoQ (Cat# MRP300) were measured using Quantikine enzyme-linked immunosorbent assay (ELISA) kits (R&D Systems, Minneapolis, MN, USA) according to the manufacturer’s instructions.

### 2.7. Quantification of Atherosclerosis

En face analysis of aortic atherosclerotic lesions was performed as described by Tangirala et al. [23]. Briefly, following perfusion fixation, the entire aorta was carefully dissected from the heart to the iliac bifurcation, opened longitudinally, and pinned flat on a black wax surface. The tissue was stained with Sudan IV solution to visualize lipid-rich lesions. Images were captured using a SONY DXC-970MD color video camera (Tokyo, Japan), and quantitative analysis was performed in a blinded manner using the Image-Pro Plus software V7.1 (Media Cybernetics, Silver Spring, MD, USA). The percentage of aortic surface area covered by atherosclerotic lesions was calculated as the ratio of lesion area to total aortic area for each specimen. Atherosclerotic lesion area in the aortic root was determined as described previously [23]. Briefly, hearts and proximal aortas were embedded in optimum cutting temperature (OCT) compound. Serial 10 μm thick cryosections extending from the middle portion of the ventricle to the aortic arch were collected, mounted on precoated slides, and stained with Oil-Red-O and hematoxylin. Lesion area surrounding the aortic valve cusps was quantified in a blinded manner using an ocular piece grid (20 × 20 μm^2^) under light microscopy. The mean aortic lesion area for each mouse was calculated from 5 to 10 serial sections.

### 2.8. Real-Time qPCR Analysis

Gene expression analysis of mouse tissues and MEFs was performed using quantitative real-time PCR (qPCR). Total RNA was isolated with TRIzol reagent (Life Technologies, Carlsbad, CA, USA). RNA concentration and purity were determined spectrophotometrically by measuring absorbance at 260 and 280 nm, and only RNA samples meeting the established purity criteria were used for subsequent analyses. In total, 2 μg of RNA was reverse-transcribed into cDNA using the High-Capacity cDNA reverse transcription kit (Applied Biosystems, Foster City, CA, USA). Quantitative real-time PCR (qPCR) was performed using Power SYBR Green PCR Master Mix (Applied Biosystems) on a 7500 Fast Real-Time PCR System (Applied Biosystems).

Reactions containing 25 ng of cDNA were run in quadruplicate, with GAPDH used as an internal control gene. Relative mRNA expression levels were quantified from standard curves and analyzed with 7500 System Software v1.4 [4,5]. Primer pairs were designed using PrimerBank and are listed in Table 1.

### 2.9. Immunoblot Analysis

Protein expression was analyzed by Western blotting. Equal amounts of protein were separated on 10–12% SDS-PAGE gels and electrotransferred onto polyvinylidene difluoride (PVDF) membranes. Membranes were blocked overnight with 10% milk, washed with 1X PBS containing 0.1% Tween 20 (PBST), and incubated with the following primary antibodies: anti-UCP1 (1:1000; Abcam, Cat# ab10983, Cambridge, MA, USA), anti-VCAM1 (1:1000; Abcam, Cat# ab134047), anti-iNOS (1:1000; Cell Signaling Technology, Cat# 13120), anti-CD68 (1:1000; Bio-Rad, Cat# FA-11), anti-TNF-α (1:1000; Santa Cruz Biotechnology, Cat# sc-52746, Dallas, TX, USA), anti-Arginase 1 (1:1000; Santa Cruz Biotechnology, Cat# sc-271430), anti-Adiponectin (AdipoQ; 1:1000; R&D Systems, Cat# AB_2305045, Minneapolis, MN, USA), anti- *Fgf21* (1:1000; R&D Systems, Cat# MAB25371), and anti-GAPDH (1:5000; Millipore, Cat# MAB374, Burlington, MA, USA) (Table 2).

Appropriate horseradish peroxidase (HRP)-conjugated secondary antibodies (1:1000; Cell Signaling Technology) were used for detection. Immunoreactive bands were visualized using enhanced chemiluminescence (ECL), and band intensities were quantified using ImageQuant software (TL 11.0, Uppsala, Sweden), as described previously [4,5].

### 2.10. Immunostaining

For immunohistochemical analysis, paraffin-embedded liver sections (5 μm) were mounted on HistoGrip-coated slides. Sections were deparaffinized in xylene, rehydrated through a graded ethanol series, and treated with 3% hydrogen peroxide (H_2_O_2_) to quench endogenous peroxidase activity. Endogenous avidin and biotin were blocked using a commercial blocking kit. Slides were incubated overnight at 4 °C in a humidified chamber with either anti-UCP1 antibody (1:100; Abcam, Cat# ab10983, Cambridge, MA, USA) or anti-Activin-A antibody (1:100; Novus Biologicals, Centennial, CO, USA; Cat# NBP1-30928). After washing with PBS, sections were incubated with a biotinylated secondary antibody for 45 min, followed by visualization using an avidin–biotin complex (ABC) peroxidase detection kit and diaminobenzidine (DAB) substrate. Slides were rinsed in tap water, counterstained with hematoxylin, and mounted using Aquatex mounting medium. Images were captured using an Olympus BX41 microscope, and the percentage of immunopositive staining area was quantified using ImageJ software version 1.54 (NIH, Bethesda, MD, USA).

For immunofluorescence staining, paraffin-embedded tissue sections were deparaffinized, and antigen retrieval was performed by boiling the sections in citrate buffer (pH 6.0; Sigma-Aldrich (St. Louis, MO, USA), Cat# 00500). Sections were then blocked for 90 min at room temperature with 10% bovine serum albumin (BSA) prepared in 1× PBST (1× PBS containing 0.1% Triton X-100). Primary antibodies included rabbit anti-Arginase 1 (1:200; GeneTex, Irvine, CA, USA), Cat# GTX10429), rat anti-CD68 (1:200; Invitrogen, Carlsbad, CA, USA), Cat# 14-0681-82), and rabbit anti-caveolin-1 (1:200; Cell Signaling Technology, Danvers, MA, USA), Cat# D46G3). Sections were incubated with primary antibodies overnight at 4 °C in a humidified chamber. After two washes with 1× PBS, slides were incubated for 90 min at room temperature with appropriate fluorescent secondary antibodies (Invitrogen, Carlsbad, CA, USA), Cat# A31573 and A11055) and counterstained with DAPI (Invitrogen, Cat# D1306). Following three PBS washes, sections were mounted using ProLong™ Gold Antifade Mount (Thermo Fisher Scientific, Waltham, MA, USA), Cat# P36931) and coverslipped. Negative controls were processed identically except that the primary antibodies were omitted. Fluorescent images were captured using a Leica DMi8 fluorescence microscope (Wetzlar, Germany) with 10× or 20× objectives, and acquisition was performed using LAS X software 5.3.1 (Wetzlar, Germany).

### 2.11. Isolation of Bone Marrow-Derived Macrophages (BMDM)

Bone marrow-derived macrophages (BMDMs) were isolated from 6 to 12-week-old C57BL/6 mice under protocols approved by the UCLA IACUC protocol #ARC-2010-003, as described previously [24,25]. Briefly, femurs and tibias were flushed with ice-cold PBS, and the resulting cell suspension was passed through a 70 µm strainer. Erythrocytes were lysed using ACK buffer. Cells were resuspended in DMEM supplemented with 10% heat-inactivated FBS, 1% penicillin-streptomycin, 2 mM L-glutamine, and recombinant M-CSF (30–50 ng/mL), and plated at 1–2 × 10^6^ cells per 10 cm dish. Cultures were maintained at 37 °C and 5% CO_2_, with medium added on day 3. On day 7, differentiated macrophages were harvested by gentle scraping, washed with PBS, and resuspended in assay medium. This protocol routinely yielded >90% CD11b^+^F4/80^+^ macrophages with >90% viability.

### 2.12. Statistical Analysis

Data are presented as mean ± SEM. Statistical analyses were performed using GraphPad Prism software (versions 5.3 or 6.0; GraphPad Software, San Diego, CA, USA). Before statistical comparisons, data were assessed for normality using the Shapiro–Wilk test and for homogeneity of variances using Levene’s test. Because the assumptions of normality and equal variance were met (Shapiro–Wilk test, *p* > 0.05; Levene’s test, *p* > 0.05), differences between groups were evaluated by one-way analysis of variance (ANOVA). When ANOVA indicated a significant overall effect, pairwise comparisons were conducted using the Newman–Keuls multiple comparison test. All statistical tests were two-tailed, and *p* ≤ 0.05 was considered statistically significant. Sample size for the murine studies was determined by a priori power analysis based on preliminary data, providing at least 80% power at α = 0.05. Each experiment was performed at least three independent times, and representative data are presented.

## 3. Results

### 3.1. Follistatin Gene Correlation with Browning and Mitochondrial Biogenesis Genes in the HMDP Dataset

We previously demonstrated that *Fst* promotes adipose tissue browning [4,5], a process that has recently been linked to attenuation of atherosclerosis progression [10,11,12]. To gain insight into the potential role of *Fst* in regulating atherosclerosis, we examined the Hybrid Mouse Diversity Panel (HMDP) dataset for correlations between *Fst* expression and genes involved in adipose browning and mitochondrial biogenesis. Several adipose browning and mitochondrial biogenesis genes were significantly and positively correlated with *Fst* expression (bicor values ranging from 0.175 to 0.378; *p* = 0.001–0.003) (Table 3A).

Among these genes, *Tmem26*, a well-known marker of adipose browning has been implicated in lipid metabolism, although its direct role in atherosclerosis remains unclear [26,27]. Cidea, expressed in brown and brite adipose tissues, regulates lipid metabolism, reduces macrophage infiltration, enhances insulin sensitivity, and contributes to metabolically healthy obesity in both humans and mice [28,29]. However, its role in atherosclerosis is largely unknown. Neuregulin 4 (Nrg4), a BAT-specific protein that activates mitochondrial function and energy metabolism, has been shown to reduce endothelial inflammation and atherosclerosis progression via ErbB4-Akt-NF-κB signaling [14,30]. Circulating Nrg4 levels are inversely associated with subclinical atherosclerosis in obese adults and in patients with acute coronary syndrome [30]. UCP3, an ortholog of UCP1 involved in energy expenditure, contributes to adipose browning and can regulate energy metabolism under specific activation conditions [31]. PGC-1α, a central regulator of mitochondrial biogenesis and oxidative metabolism, also plays a protective role in atherosclerosis [32,33]. Overexpression of PGC-1α in human aortic smooth muscle cells (HASMCs) and endothelial cells (HAECs) reverses TNF-α-induced endothelial injury and suppresses NF-κB activity, reducing MCP-1 and VCAM-1 expression and thereby preventing lesion development [34]. Human atherosclerotic lesions exhibit reduced PGC-1α expression in symptomatic compared to asymptomatic plaques, while PGC-1α overexpression via conjugated linoleic acid treatment inhibits macrophage foam cell formation by limiting oxidized lipid uptake [35]. Cox8a, a mitochondrial gene involved in oxidative phosphorylation, has been implicated in protection against atherosclerosis progression. Gene set enrichment analysis of atherosclerotic mouse aorta microarray data revealed significant downregulation of Cox8a expression compared with controls [36]. Collectively, these genes were positively correlated with *Fst* expression in HMDP dataset and are known regulators of mitochondrial biogenesis and adipose browning. Notably, these genes were also found to be significantly upregulated by *Fst* in our previous in vitro and in vivo studies [4,5].

Next, we analyzed the correlation of *Fst* expression with aortic lesion area in the HMDP dataset (Table 3B). In female liver, *Fst* expression showed a negative trend but did not reach statistical significance. However, *Fst* expression in the female aorta was significantly and negatively correlated with aortic lesion area (r = −0.251; *p* = 0.03), ranking higher in significance than *Fgf21* (r = −0.206; *p* = 0.07 in male liver; r = −0.183; *p* = 0.075 in female liver) a well-known modulator of atherosclerosis. These findings support a potential anti-atherogenic role for *Fst*.

### 3.2. Follistatin Gene Delivery Inhibits Atherosclerotic Lesion Formation and Improves Plasma Lipid Levels in Ldlr^−/−^ Mice Fed a Western Diet

**Figure 1:**
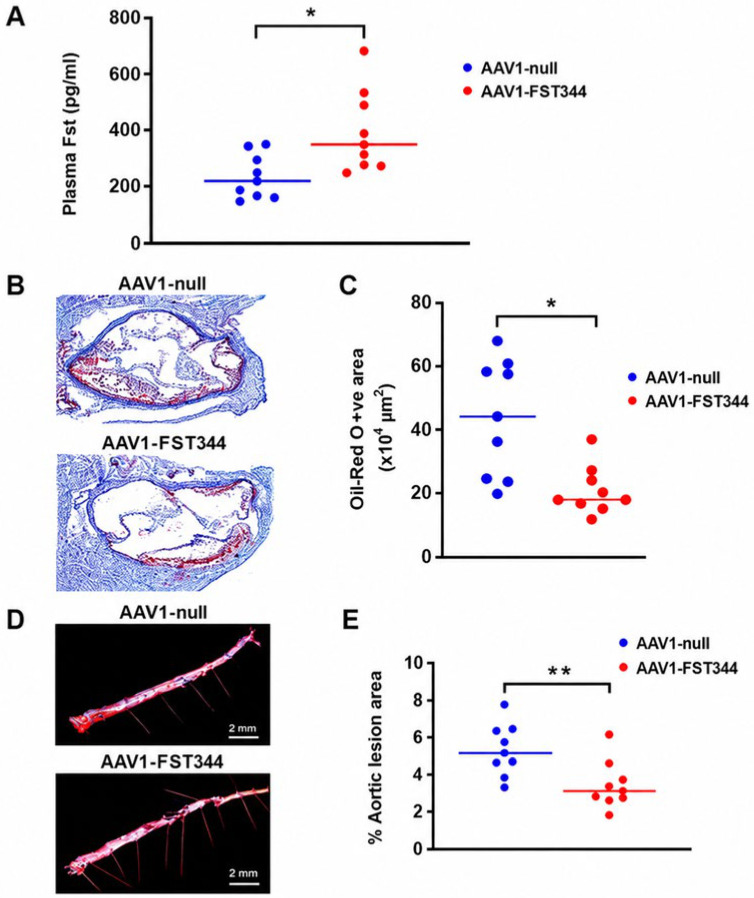
(**A**): Effect of AAV1-FST344 gene delivery on plasma Fst levels and atherosclerotic lesions. Eight-week-old *Ldlr*^−/−^ male mice were injected via tail vein with 5 × 10^11^ viral particles of AAV1-FST344 or AAV1-null and fed a WD for 12 weeks on western diet. (**A**) Plasma Fst levels measured by ELISA. (**B**) Oil-Red-O staining of the heart. (**C**) Quantitative analysis of Oil-Red-O-stained heart sections. (**D**) Sudan IV staining of the aortic lesion. (**E**) Quantitative analysis of aortic lesion area. Data are presented as mean ± SEM. *, *p* ≤ 0.05; **, *p* ≤ 0.01 (*n* = 9 mice each group).

To investigate the effect of exogenous follistatin on atherosclerosis, 8-week-old male *Ldlr*^−/−^ mice were injected via tail vein with 5 × 10^11^ viral particles of AAV1-FST344 or control AAV1-null vector and subsequently fed a WD for 12 weeks. FST344, the predominant secreted splice variant of follistatin with an extended circulating half-life, has been shown to be efficiently expressed and secreted, enabling effective gene delivery in multiple animal models [37,38]. Consistent with effective gene delivery, plasma follistatin levels measured by ELISA were markedly increased in the FST344 group compared with controls (419 ± 384 vs. 203 ± 47 ng/mL; *p* = 0.017) (Figure 1A). Circulating levels of follistatin in WT and AAV1-null groups were not significantly different (211± 65 vs. 203 ± 58 ng/mL) after viral treatment, indicating that the AAV1 intervention did not significantly elevate systemic FST beyond baseline physiological levels in the control condition. No significant differences in body weight were observed between groups (Figure S1), although food intake was modestly decreased in the AAV1-FST344 group compared with the control (Figure S2). Although food intake was modestly reduced following AAV1-FST344 treatment (Figure S2), body weight remained unchanged (Figure S1), likely because the increase in lean muscle mass was offset by a reduction in adipose tissue mass. The lesion area in the aortic root as determined by Oil-Red-O staining shows significantly reduced atherosclerotic lesions in the FST344-injected mice compared with AAV1-null controls (Figure 1B). Quantitative analysis of the Oil-Red-O positive lesion areas in the aortic root sections show significant decrease in the FST344 group compared with the control (20.9 ± 8.4 × 10^4^ µm^2^ vs. 45.3 ± 24.4 × 10^4^ µm^2^; *p* = 0.04) (Figure 1C). En face analysis of whole aortas using Sudan IV staining confirmed a significant reduction in lesion area in the FST344 group (3.5 ± 0.6% vs. 5.4 ± 1.9%; *p* = 0.009) (Figure 1D,E). Liver triglyceride (TG) content was also significantly decreased following AAV1-FST344 treatment (91.8 ± 42.1 vs. 139.6 ± 42.1 mg/dL; Figure S3), indicating an improvement in hepatic lipid metabolism and reduced hepatic lipid accumulation. Plasma lipid analysis demonstrated significant reductions in triglycerides (TG), and free-fatty-acids (FFA), together with a significant increase in high-density lipoprotein (HDL) levels (Table 4).

### 3.3. Fst Gene Delivery Promotes Adipose Browning and Suppresses Adhesion and Inflammatory Markers in WAT, Liver, and Heart of Ldlr^−/−^ Mice Fed a Western Diet

To further validate the HMDP gene correlation analyses and determine whether FST regulates additional thermogenic genes, we measured the expression of beige adipocyte and inflammation markers in WAT, liver, and heart from AAV1-FST344 and control-AAV1-null vector treated mice.

**Figure 2:**
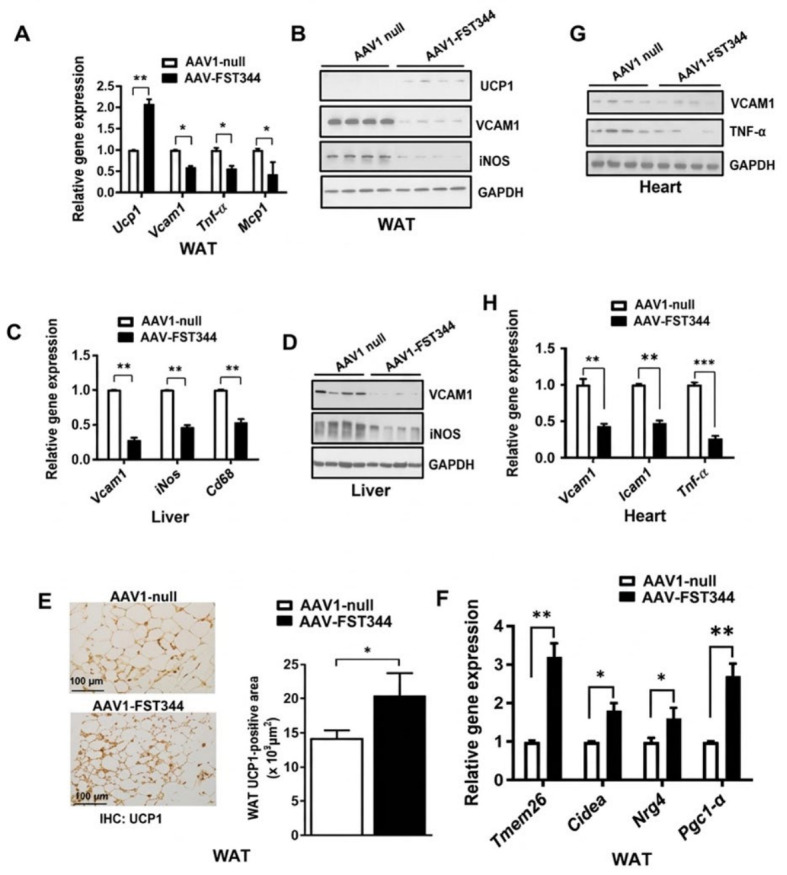
Effect of AAV1-FST344 gene delivery on adipose browning and markers of adhesion and inflammation in *Ldlr*^−/−^ mice fed a WD for 12 weeks. (**A**) Quantitative RT-PCR analysis of genes associated with adipose browning (*Ucp1*), adhesion (*Vcam1*), and inflammation (*Tnf-α* and *Mcp1*), (n = 3). (**B**) Representative Western blot analysis of UCP1, VCAM, and iNOS proteins in WAT, (n = 3). (**C**) Quantitative RT-PCR analysis of *Vcam1*, *iNos*, and macrophage (*Cd68*)-related genes, (n = 3). (**D**) Representative Western blot analysis of VCAM1 and iNOS proteins in liver, (n = 3). (**E**) (**Left**): Immunohistochemistry of WAT using anti-UCP1 antibody in AAV1-null and AAV1-FST344 groups. (**Right**): Quantification of UCP1-positive area (* *p* ≤ 0.05), (n = 6). (**F**) Quantitative RT-PCR analysis of additional adipose browning genes to validate the HMDP dataset correlation of *Fst* gene with mitochondrial biogenesis genes as shown in Table 3A. (n = 3) (**G**) Western blot analysis of VCAM1 and TNF-α proteins in heart tissues from AAV1-null and AAV1-FST344 mice. (n = 3) (**H**) Quantitative RT-PCR analysis of *Vcam1*, *Icam1* and, *Tnf-α* genes in hearts; **, *p* ≤ 0.01; ***, *p* ≤ 0.001, (n = 3).

In WAT, both gene and protein expression of uncoupling protein 1 (UCP1), a key marker of beige adipocytes, were significantly increased in the FST344 group compared with controls (Figure 2A,B,E). Concurrently, expression of genes encoding adhesion molecules (*Vcam1*, *Mcp1*, *Icam1*), inflammatory mediators (*Tnf-α*, *iNos*), and macrophage marker *Cd68* were significantly downregulated in WAT, liver, and heart tissues following *Fst* gene delivery (Figure 2A,C,H).

Consistent with transcriptional changes, protein levels of VCAM1, iNOS, and TNF-α were also markedly decreased in these tissues after AAV1-FST344 treatment (Figure 2B,D,G). To further test the effect of *Fst* on additional beige markers as well as validate the observed *Fst* gene correlation with mitochondrial biogenesis markers from the HMDP dataset, we compared gene expression levels of these beige and mitochondrial genes expression levels in WAT obtained from AAV-1-null and AAV1-FST344 groups. Expression of *Tmem26*, *Cidea*, *Nrg4*, as well as *Pgc1-α* genes significantly increased in WAT from AAV1-FST344 treated mice compared with control mice (Figure 2F).

Together, these findings indicate that *Fst* gene delivery in *Ldlr*^−/−^ mice fed a WD promotes adipose browning while simultaneously suppressing adhesion and inflammatory markers, as well as macrophage recruitment, across key metabolic tissues implicated in atherosclerosis progression.

### 3.4. Aortic Lesion Area Correlates with Plasma Arginine, Citrulline, and Ornithine Levels in HMDP Mice

Correlation analysis of aortic lesion area with plasma metabolites in HMDP mice identified several key components of the arginine metabolic pathway, including arginine, citrulline, and ornithine, among others. Plasma levels of arginine (bicor = 0.316; *p* = 0.0146) and citrulline (bicor = 0.304; *p* = 0.0192) showed strong positive correlations with aortic lesion area, whereas ornithine levels were negatively correlated (bicor = −0.402; *p* = 0.0016) (Table 5).

**Figure 3:**
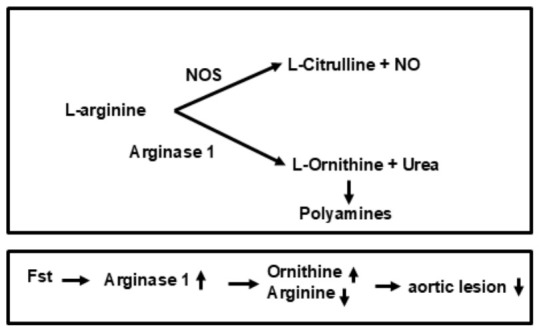
L-arginine metabolism and the hypothetical role of *Fst* in atherosclerosis. Top: Schematic representation of L-arginine metabolic pathways. Bottom: Hypothetical mechanism by which *Fst* promotes Arg1-catalyzed conversion of L-arginine to L-ornithine, potentially reducing aortic lesion formation, based on correlations observed in the HMDP dataset.

These correlations suggested that anti-atherogenic effects of *Fst* may be mediated, at least in part, through modulation of arginine metabolism, characterized by reduced arginine and citrulline levels together with increased ornithine production. These findings suggest a potential involvement of the arginase 1 (Arg1)-catalyzed metabolic pathway in *Fst*-induced anti-atherogenic action. We propose that activation of Arg1 by *Fst* promotes the conversion of L-arginine to L-ornithine, thereby contributing to decreased aortic lesion area and attenuation of atherosclerosis progression (Figure 3).

### 3.5. Expression Correlates with Aorta and Liver Arginine and Ornithine Levels in the HMDP Dataset

To investigate key metabolic pathways potentially mediating *Fst*-induced anti-atherogenic effects, we analyzed the HMDP metabolomic dataset for correlations between *Fst* gene expression and tissue metabolites in aorta and liver. We observed strong positive correlations between *Fst* expression and ornithine levels in both aorta (bicor = 0.4116; *p* = 0.0002) and liver (bicor = 0.3853; *p* = 0.0026), and a significant negative correlation with arginine levels in liver (bicor = −0.3372; *p* = 0.009) (Table 6). Additionally, branched-chain amino acids (leucine, isoleucine, and valine) were positively correlated with *Fst* expression in these tissues (Table 6). Phenylalanine levels also correlated positively with *Fst* expression in both liver and aorta. As a precursor of catecholamines, phenylalanine has been reported to be inversely associated with atherosclerotic lesion area [39]. Collectively, these findings suggest that Fst contributes to anti-atherogenic process through coordinated regulation of arginine-ornithine metabolism and other amino acid metabolic pathways in vascular and metabolic tissues.

### 3.6. AAV1-FST344 Significantly Upregulates Arginase 1 and Fgf21 in Adipose, Liver, and Heart Tissues of Ldlr^−/−^ Mice Fed a Western Diet

**Figure 4:**
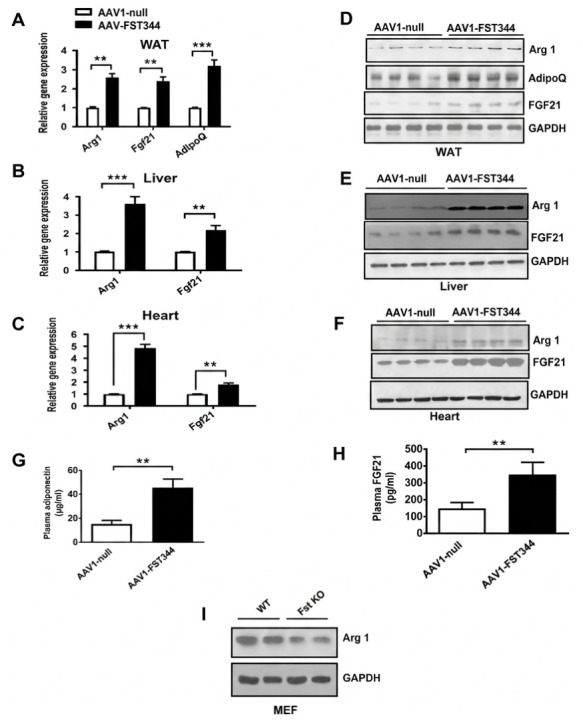
Effect of AAV1-FST344 gene delivery on tissue Arg1, *Fgf21*, and AdipoQ expression in WAT, liver, and heart in *Ldlr*^−/−^ mice fed a WD for 12 weeks. (**A**–**C**) Quantitative RT-PCR analysis of *Arg1* and *Fgf21* gene expression in WAT, liver, and heart respectively (n = 3). (**D**–**F**) Representative Western blots showing Arg1 and *Fgf21* protein expression in WAT, liver, and heart tissues respectively, (n = 3). (**G**) Plasma adiponectin and (**H**) *Fgf21* levels in AAV1-null and AAV1-FST344 groups, (n = 9). (**I**) Western blot of Arg1 protein in WT and *Fst* KO MEF primary cultures. Data are presented as mean ± SEM (n = 6–9). **, *p* ≤ 0.01; ***, *p* ≤ 0.001, (n = 3).

Based on HMDP gene–metabolite correlation analyses implicating arginine metabolism in FST-mediated vascular protection, we examined expression of Arg1 and *Fgf21* in *Ldlr*^−/−^ mice fed a WD. Gene (Figure 4A–C) and protein (Figure 4D–F) expression analyses revealed that AAV1-FST344-mediated follistatin gene delivery significantly upregulated *Arg1* (WAT: ~2.6-fold; liver: ~3.6-fold; heart: ~4.86-fold) and *Fgf21* (WAT: ~2.4-fold; liver: ~2.2-fold; heart: ~1.82-fold) compared with control vector-treated mice. *AdipoQ*, an adipose-specific gene implicated in metabolic regulation and reported to inhibit atherosclerosis either independently or in cooperation with *Fgf21* [40,41], was also significantly increased (~3.2-fold) following follistatin delivery in WAT (Figure 4A). Protein expression analyses confirmed upregulation of *Arg1*, *Fgf21*, and *AdipoQ* in these tissues following AAV1-FST344 treatment (Figure 4D–F). Plasma measurements further demonstrated significantly elevated adiponectin (~3-fold) and *Fgf21* (~2.4-fold) levels in AAV1-FST344-treated mice compared to controls (Figure 4G,H). Consistent with these *in vivo* findings, Arg1 protein expression was significantly reduced in *Fst* knockout (KO) MEF cultures compared with wild-type (WT) littermate controls (Figure 4I). Comparative gene expression analysis of *Fst* KO versus WT MEFs differentiated under adipogenic conditions [4,5] further showed decreased expression of *Arg1*, *AdipoQ*, *Ucp1*, and *Fgf21* genes in *Fst* KO MEF cultures (Figure S4), suggesting that follistatin deficiency impairs the expression of genes associated with adipocyte differentiation, thermogenesis, and metabolic regulation. In an *Fst* transgenic mouse model (*Fst*-Tg) [5], liver and plasma concentrations of ornithine and urea, key products of Arg1-catalyzed metabolism, were significantly higher than in WT mice (Figure S5A–D), suggesting that follistatin promotes Arg1-mediated arginine catabolism, a pathway implicated in metabolic regulation and immune modulation that may represent a mechanistic link between follistatin signaling and the attenuation of atherosclerotic disease. Collectively, these findings demonstrate that FST robustly induces Arg1 expression in multiple in vitro and in vivo models, supporting a central role for Arg1 in mediating the anti-atherogenic action of FST.

### 3.7. Co-Localization of Arginase 1 and CD68 in Heart and Liver Tissues of Null and FST344-Injected Mice

**Figure 5:**
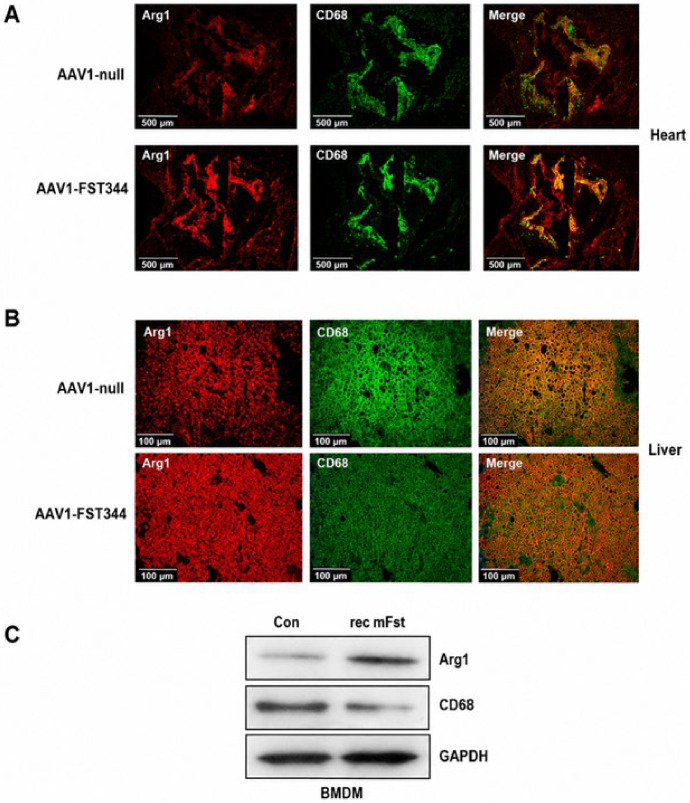
Double immunofluorescence analysis of (**A**) heart and (**B**) liver sections from null and FST344-injected *Ldlr*^−/−^ mice fed a WD for 12 weeks. Sections were stained with anti-Arg1 (red) and anti-CD68 (green) antibodies. Co-localization of Arg1 and CD68 is indicated by yellow in merged images, highlighting macrophage-associated Arg1 expression, (*n* = 4–6). (**C**) Effect of recombinant mouse *Fst* 288 (rec mFST) (0.5 μg/mL) on BMDM primary cultures isolated from 6-week-old C57BL6/J male mice, (*n* = 3).

Building on our previous findings of significant Arg1 upregulation in WAT, liver, and heart following AAV1-FST344 injection in *Ldlr*^−/−^ mice and the observed decrease in Arg1 expression in *Fst* KO MEFs, we next examined whether the increased Arg1 expression following *Fst* gene delivery localized to tissue macrophages [42,43]. Heart and liver tissues from AAV1-null-treated *Ldlr*^−/−^ mice fed a WD for 12 weeks showed detectable Arg1 (red) and CD68 (green) expression (Figure 5A,B). Follistatin delivery via AAV1-FST344 markedly increased Arg1 expression while simultaneously reducing CD68 levels in both tissues (Figure 5A,B). Merged images revealed significant co-localization of Arg1 and CD68 (yellow), suggesting that follistatin modulates Arg1 and macrophage marker expression in a reciprocal manner. In the context of atherosclerosis, elevated Arg1 expression in macrophages is typically associated with an anti-inflammatory, M2-like phenotype, which promotes tissue repair and plaque stabilization [42,43]. These findings suggest that FST promotes macrophage polarization towards anti-inflammatory phenotype characterized by elevated Arg1 expression. We next evaluated the effect of *Fst* on Arg1 and CD68 expression in primary bone marrow-derived macrophages (BMDMs) isolated from male C57BL/6 mice (Figure 5C). Treatment with 0.5 μg/mL recombinant *Fst*288 (r*Fst*288) increased Arg1 protein levels while concomitantly reducing CD68 expression after 48 h of treatment. These findings indicate that *Fst* mediates reciprocal regulation of Arg1 and CD68 expression in macrophages.

### 3.8. FST344 Reduces Caveolin-1 Expression in Liver and Adipose Tissues

**Figure 6:**
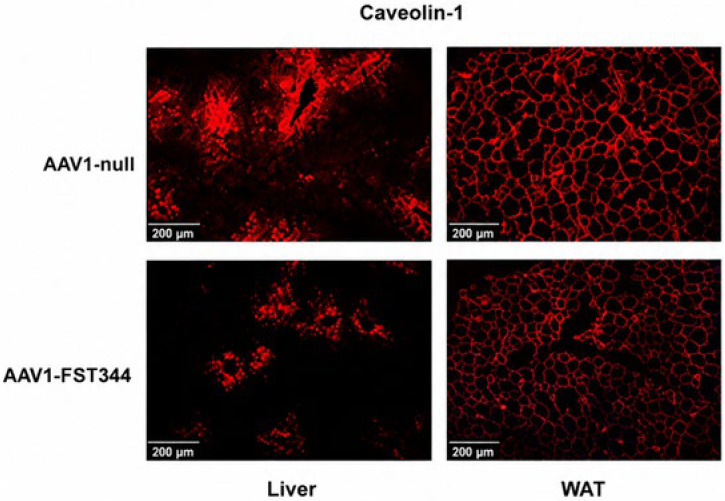
Caveolin-1 expression in liver and epididymal WAT sections from AAV1-null and AAV1-FST344-injected *Ldlr*^−/−^ mice fed a WD for 12 weeks. Sections were stained with anti-Cav1 antibody (red), (n = 4).

Caveolin-1 plays a key role in mediating LDL transport across endothelial cells, a critical step in atherosclerotic plaque formation [44]. The absence of caveolin-1 has been associated with reduced lipid accumulation and inflammation in atherosclerotic lesions, highlighting its pro-atherogenic role [44,45]. We examined caveolin-1 expressions in liver and epididymal WAT from AAV1-null and AAV1-FST344-treated mice fed a WD for 12 weeks. FST344 delivery significantly decreased caveolin-1 expression in both tissues compared with control mice (Figure 6). These findings suggest that FST attenuates atherogenesis, at least in part, by suppressing caveolin-1-mediated lipid transport.

### 3.9. FST344 Downregulates Activin-A Expression in Liver and Adipose Tissues

**Figure 7:**
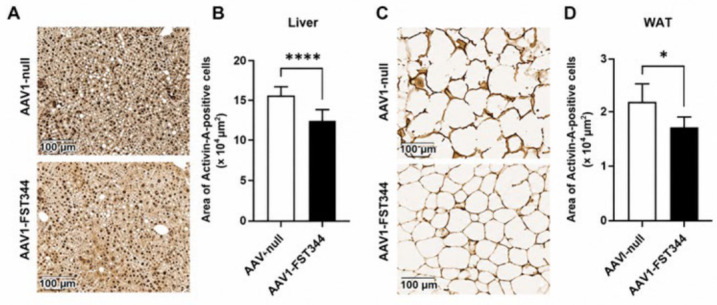
Activin-A expression in liver and epididymal WAT sections from AAV1-null and AAV1-FST344-injected *Ldlr*^−/−^ mice fed a WD for 12 weeks. (**A**,**C**): Representative immunohistochemistry of liver and WAT using anti-Activin-A antibody in AAV1-null and AAV1-FST344 groups. (**B**,**D**): Quantification of Activin-A-positive area (****, *p* ≤ 0.001; * *p* ≤ 0.05), (*n* = 6–8).

Given that Activin-A, a member of the TGF-β superfamily, is upregulated in atherosclerotic lesions and contributes to vascular inflammation, fibrosis, and plaque remodeling, we investigated the effect of FST344 on Activin-A expression liver and adipose tissues collected from in *Ldlr*^−/−^ mice. Activin-A expression was significantly reduced in both liver and WAT following AAV1-FST344 treatment compared with AAV1-null control (Figure 7A–D). The ability of FST344 to neutralize Activin-A signaling is particularly relevant in the context of atherosclerosis, where increased Activin-A expression has been associated with vascular inflammation and lesion development. These findings identify FST as an endogenous antagonist of Activin-A signaling that may contribute to vascular protection by suppressing pro-inflammatory and pro-fibrotic TGF-β superfamily signaling.

## 4. Discussion

Atherosclerosis, a chronic inflammatory disease characterized by the accumulation of lipids and fibrous elements in the arterial wall, remains a leading cause of morbidity and mortality worldwide. Our previous studies identified FST, a known inhibitor of TGF-β signaling, as a novel inducer of adipose browning that regulates distinct molecular pathways promoting brown adipose characteristics, improving circulatory lipoprotein profiles, and favorably modulating metabolites implicated in lipid and cholesterol metabolism [4,5]. Increased energy expenditure by brown adipose tissue (BAT) plays a critical role in TG and low-density lipoprotein cholesterol (LDL-C) clearance and confers atheroprotective effects [46,47]. In this study, we investigated the anti-atherogenic potential of FST and sought to identify cellular and molecular mechanisms underlying its protective effects. Previous work reported the in vivo expression of Activin-A and its endogenous inhibitor FST in human vascular tissue at different stages of atherosclerosis, as well as in macrophages, smooth muscle cells, and endothelial cells in vitro, suggesting that the Activin–A/FST axis plays a significant role in atherogenesis [48,49]. While Activin-A promotes pro-inflammatory and pro-atherogenic signaling in the vascular wall, the protective role of FST may involve binding to and inhibition of Activin-A; however, conclusive evidence remains lacking.

The HMDP dataset provides a powerful resource for identifying genetic and metabolic determinants of complex traits such as atherosclerosis [19,20]. Using this dataset, we examined *Fst* gene correlations with mitochondrial biogenesis and adipose browning genes, as well as aortic lesion areas in liver and aorta tissues. Several key genes including *Pgc-1α*, *Cox8a*, *Nrg4*, *Tmem26*, and *Cidea* were positively correlated (bicor = 0.17–0.38) with *Fst* expression, suggesting a link between follistatin, adipose browning, and mitochondrial function (Table 3A). Importantly, *Fst* gene expression negatively correlated with aortic lesion area in female mice (Figure 1B). For the first time, our data provide strong evidence of the anti-atherogenic function of viral vector-mediated *Fst* gene delivery in *Ldlr*^−/−^ mice, demonstrated by significantly reduced lesion areas and improved plasma lipid profiles compared to control mice. These changes were accompanied by upregulation of UCP1, a key browning marker in WAT, and downregulation of adhesion (VCAM1, ICAM1, MCP1) and inflammatory (TNF-α, iNOS) markers in WAT, liver, and heart tissues, highlighting a critical role for FST-induced browning in its protective effects. These findings align with prior studies showing that β3-adrenergic receptor (β3-AR) activation, via cold exposure or agonist CL316243, reduces plasma TG levels and atherosclerosis through adipose browning [10]. In *Fst*-transgenic mice, CL316243 further enhances thermogenic responses, further supporting the role of FST in regulating browning and energy metabolism [5].

Arginine metabolism emerged as another key pathway influenced by FST. Correlation analyses from the HMDP dataset revealed that plasma arginine and citrulline positively correlate with aortic lesion area, whereas ornithine exhibited a negative correlation, suggesting that Arg1-mediated conversion of arginine to ornithine may contribute to atheroprotection (Figure 4, Table 4 and Table 5). Consistent with these observations, *Fst*-transgenic mice displayed elevated liver and plasma ornithine and urea concentrations compared with wild-type controls (Figure S5), while AAV1-FST344 increased Arg1 expression in WAT, liver, and heart of *Ldlr*^−/−^ mice. In contrast, Arg1 expression was reduced in *Fst*-KO MEF cultures, suggesting that FST is a possible upstream driver of Arg1 expression in adipose browning. Arg1 is a well-recognized marker of anti-inflammatory M2 macrophages, which promote tissue repair and are inversely associated with atherosclerosis progression [42,43,50,51]. Mechanistically, Arg1 may protect against atherosclerosis by limiting nitric oxide (NO) production via inducible nitric oxide synthase (iNOS), promoting collagen synthesis, supporting efferocytosis, and stabilizing the fibrous cap of lesions [51]. Previous studies in rabbits, mice, and humans have demonstrated that increased macrophage Arg1 expression reduces lesion size, inflammation, and foam cell formation, whereas Arg1 deficiency exacerbates atherosclerosis progression [52,53,54]. Our data suggest that FST contributes to atheroprotection, at least in part, through induction of Arg1, increased ornithine production, and enhanced macrophage-mediated tissue repair. While the precise mechanism by which FST regulates Arg1 remains unclear, several transcriptional regulators including RXR/PPARδ/γ, IL4/IL10/PU.1, IL6/pSTAT3, IRF8, LXRα, cFos/Jun, and Nur77/VDR have been implicated in regulating Arg1 transcription [53,54,55,56,57,58]. The increased IL6/pSTAT3 signaling observed following FST overexpression further supports the potential involvement of this regulatory network [59,60]. In addition to Arg1, FST increased *Fgf21* expression in WAT, liver, and heart, while also elevating circulating *Fgf21* and adiponectin levels [59]. The *Fgf21*–adiponectin endocrine axis has been widely implicated in protection against atherosclerosis and other cardio-metabolic disorders, through coordinated multiorgan crosstalk among liver, adipose tissue, and vasculature [40,41,61]. Moreover, β3-AR activation stimulates *Fgf21* transcription via PKA/p38 MAPK signaling, reinforcing adipose browning and metabolic adaptation [62,63]. These findings further support the concept that FST regulates multiple interconnected metabolic pathways that collectively improve systemic metabolic homeostasis. Our results suggest that FST exerts both direct tissue-specific effects and indirect systemic metabolic effects. Systemic AAV-mediated *Fst* gene delivery resulted in broad remodeling of metabolic and inflammatory pathways in the liver, heart, and WAT, including increased expression of key regulators such as Arg1 and *Fgf21*. The co-localization of Arg1 within CD68^+^ macrophages further indicates that macrophages represent an important cellular target mediating the anti-inflammatory effects of FST.

Direct actions of FST likely include modulation of adipocyte metabolism and browning, regulation of hepatic metabolic signaling and *Fgf21* production, and polarization of macrophages toward an anti-inflammatory Arg1^+^ phenotype through inhibition of Activin-A and TGF-β signaling. However, because FST was delivered systemically, some of the observed molecular and cellular changes are likely secondary to overall metabolic improvement including reduced lipid accumulation, and improved lipid homeostasis. Consequently, although our findings support both direct and indirect mechanisms of FST action, the relative contribution of each pathway cannot be fully distinguished and will require future cell type-specific mechanistic studies. Furthermore, our findings suggest that follistatin coordinates an integrated network involving adipose tissue browning, macrophage Arg1 induction, and *Fgf21* signaling. Through antagonism of Activin-A/TGF-β pathways, FST may promote thermogenic remodeling and improve systemic metabolic efficiency, while concurrently driving an anti-inflammatory, Arg1-enriched macrophage phenotype that supports tissue repair and beige adipocyte formation. In parallel, *Fgf21* likely acts as an endocrine mediator that amplifies fatty acid oxidation, thermogenesis, and insulin sensitivity.

Collectively, our findings support a model in which FST coordinates an integrated immune–metabolic network involving adipose tissue browning, macrophage Arg1 induction, and FG21 signaling. However, the precise hierarchy and causal relationships among these components remain to be defined. Future studies will characterize the binding affinity and kinetics of mutant FST toward TGF-β family ligands using cell-based assays and surface plasmon resonance (SPR) to provide further mechanistic insights of FST-mediated protection against atherosclerosis. Consistent with our findings in *Ldlr*^−/−^ mice, unpublished observations from *apoE*^−/−^ female mice fed a WD for 14 weeks showed significantly lower plasma FST levels than mice maintained on a normal diet, and FST expression in the aorta was localized predominantly to CD68^+^ macrophage-rich regions within atherosclerotic lesions. These unpublished observations in *apoE*^−/−^ mice are consistent with our findings in *Ldlr*^−/−^ mice and further support a potential endogenous anti-atherogenic role for FST during atherosclerosis development.

## 5. Conclusions

In conclusion, our study identifies FST as a versatile regulator with strong anti-atherogenic potential, acting through integrated metabolic and anti-inflammatory pathways. FST promotes adipose browning and mitochondrial biogenesis, leading to increased energy expenditure and enhanced lipid clearance, thereby improving systemic lipid profiles. In parallel, FST reshapes arginine metabolism by upregulating Arg1, promoting ornithine production and thereby reducing inflammation, enhancing efferocytosis, and contributing to plaque stabilization. FST also activates the *Fgf21*-adiponectin axis, supporting coordinated metabolic and vascular protection across tissues. These beneficial effects are further reinforced by decreased expression of adhesion molecules and pro-inflammatory markers, which limit macrophage infiltration and vascular inflammation. Collectively, these findings establish FST as a promising therapeutic target for atherosclerosis and provide strong rationale for further studies to further elucidate its underlying mechanisms and evaluate its translational potential.

## Figures and Tables

**Table 1:** Primer sequences used for real-time quantitative PCR analysis.

Primer GenBank Accession	Forward (5′-3′)	Reverse (5′-3′)
*Ucp1* NM_009463	GTGAACCCGACAACTTCCGAA	TGAAACTCCGGCTGAGAAGAT
*Vacm1* NM_01693	TTGGGAGCCTCAACGGTACT	GCAATCGTTTTGTATTCAGGGGA
*Icam1* NM_010493	GTGATGCTCAGGTATCCATCCA	ACAGTTCTCAAAGCACAGCG
*Tnf*-α NM_013693	CAGGCGGTGCCTATGTCTC	CGATCACCCCGAAGTTCAGTAG
*Mcp1* NM_011333	TAAAAACCTGGATCGGAACCAAA	GCATTAGCTTCAGATTTACGGGT
*iNOS* NM_010927	GTTCTCAGCCCAACAATACAAGA	GTGGACGGGTCGATGTCAC
*Cd68* NM_009853	TGTCTGATCTTGCTAGGACCG	GAGAGTAACGGCCTTTTTGTGA
*Arg 1* NM_007482	CTCCAAGCCAAAGTCCTTAGAG	GGAGCTGTCATTAGGGACATCA
*Fgf21* NM_020013	GTGTCAAAGCCTCTAGGTTTCTT	GGTACACATTGTAACCGTCCTC
*AdipoQ* NM_009605	TGTTCCTCTTAATCCTGCCCA	CCAACCTGCACAAGTTCCCTT
*Tmem26* NM_177794	GAAACCAGTATTGCAGCACCC	CCCATTCCATTGGTGGCTCT
*Cidea* NM_007702	ATCACAACTGGCCTGGTTACG	TACTACCCGGTGTCCATTTCT
*Pgc1*-α NM_008904	TATGGAGTGACATAGAGTGTGCT	CTGGGCAAAGAGGCTGGTC
*Nrg4* NM_032002	TCCTCCTCACTCTTACCATCGC	GTCTCTACCAGGCTGATCTCAC
*Gapdh* NM_008084	AGGTCGGTGTGAACGGATTTG	GGGGTCGTTGATGGCAACA

**Table 2:** List of antibodies used in this study (WB: Western blot; IHC: Immunohistochemistry; IF: Immunofluorescence).

Antibody	Dilution	Catalog #	Manufacturer
anti-UCP1	1:1000 (WB) 1:100 (IHC)	ab10983	Abcam, Cambridge, MA, USA
anti-VCAM1	1:1000 (WB)	ab134047	Abcam, Cambridge, MA, USA
anti-iNOS	1:1000 (WB)	13120	Cell Signaling Technology, Danvers, MA, USA
anti-CD68	1:1000 (WB) 1:100 (IF)	FA-11	Bio-Rad Laboratories, Hercules, CA, USA
anti-TNF-α	1:1000 (WB)	sc-52746	Santa Cruz Biotechnology, Dallas, TX, USA
anti-Arginase 1	1:1000 (WB) 1:100 (IF)	sc-271430	Santa Cruz Biotechnology, Dallas, TX, USA
anti-Adiponectin (ADIPOQ)	1:1000 (WB)	MAB1119	R&D Systems, Minneapolis, MN, USA
anti- *Fgf21*	1:1000 (WB)	MAB25371	R&D Systems, Minneapolis, MN, USA
anti-Activin-A	1:100 (IHC)	NBP1-30928	Novus Biologicals, Centennial, CO, USA
anti-GAPDH	1:3000 (WB)	sc-32233	Santa Cruz Biotechnology, Dallas, TX, USA

**Table 3:** (**A**) *Fst* gene correlation with mitochondrial biogenesis genes in HMDP dataset. (**B**) *Fst* and *Fgf21* gene correlation with aortic lesion area in HMDP dataset. * *p* ≤ 0.05.

(**A**)				
**Dataset**	**Tissue**	**Gene**	**Bicor**	***p*-Value**
Male	Liver	*Tmem26*	0.306	0.002
Female	Liver	*Cidea*	0.175	0.009
Female	aorta	*Nrg4*	0.359	0.002
Female	aorta	*Ucp3*	0.359	0.001
Female	aorta	*Pgc1* *α*	0.378	0.001
Female	aorta	*Cox8a*	0.343	0.003
(**B**)				
**Dataset**	**Tissue**	**Gene**	**Biocor**	***p*-Value**
Female	aorta	*Fst*	−0.251	0.0312 *
Female	liver	*Fst*	−0.165	0.109
Male	liver	*Fst*	0.025	0.83
Male	liver	*Fgf21*	−0.206	0.072
Female	liver	*Fgf21*	−0.183	0.075
Female	aorta	*Fgf21*	0.042	0.725

**Table 4:** Plasma lipoprotein levels in *Ldlr*^−/−^ mice injected with AAV1 viral vectors (*n* = 9 mice each group).

	*Ldlr*^−/−^ + AAV1-Null	*Ldlr*^−/−^ + AAV1-FST344	*p* Value
TG (mg/dL)	211.1 ± 30.83	55.40 ± 10.49	0.02
TC (mg/dL)	1876 ± 473	1403 ± 212	0.04
HDL (mg/dL)	65.7 ± 4.8	102.9 ± 18.6	0.005
FFA (mg/dL)	57.6 ± 1.6	45.1 ± 1.8	0.02

Collectively, these results indicate that *Fst* gene delivery attenuates atherosclerotic lesion formation and improves plasma lipid profiles, supporting a potential anti-atherogenic role for *Fst* in *Ldlr*^−/−^ mice.

**Table 5:** Plasma metabolite correlation with aortic lesion.

Plasma Metabolite	Correlation (r)	*p* Value
arginine	0.316	0.0146
butylcarnitine	−0.281	0.0309
citrulline	0.304	0.0192
trimethylamnine	0.289	0.0058
ornithine	−0.402	0.0016

**Table 6:** *Fst* gene metabolite correlation in aorta and liver tissues in the HMDP dataset.

Gene	Aorta Metabolite	Correlation	*p* Value	Gene	Liver Metabolite	Correlation	*p* Value
*Fst*	isoleucine	0.5198	6.62 × 10^−5^	*Fst*	ornithine	0.3853	0.0026
	leucine	0.5192	6.79 × 10^−5^		isoleucine	0.3565	0.0056
	crotonobetaine	0.5122	8.82 × 10^−5^		leucine	0.3554	0.0057
	butyrobetaine	0.5129	0.0002		arginine	−0.3372	0.0090
	ornithine	0.4116	0.0002		valine	0.2915	0.0251
	acetyl-carnitine	0.3710	0.0044		phenylalanine	0.2904	0.0250
	phenylalanine	0.3205	0.0429				

## Data Availability

All data generated or analyzed during this study are included in this published article.

## Supplementary Materials

The following supporting information can be downloaded at: https://www.mdpi.com/article/10.3390/cells15131205/s1, Figure S1: Weekly body weight analysis. 8-week-old Ldlr^−/−^ male mice were injected via tail vein with 5 × 10^11^ viral particles of either AAV1-null or AAV1-FST344 or AAV1-null and fed a WD for 12 weeks. *n* = 8–9.; Figure S2: Analysis of weekly food consumption. 8-week old Ldlr^−/−^ male mice were injected via tail vein with 5 × 10^11^ viral particles of either AAV1-null or AAV1-FST344 or AAV1-null and fed a WD for 12 weeks. *n* = 8–9; *, *p* = 0.04; Figure S3:Analysis of liver TG levels in Ldlr^−/−^ male mice. Mouse liver triglycerides were quantified using a commercial colorimetric assay kit (Abcam, ab65336) according to the manufacturer’s instructions, in which liver tissue was homogenized, triglycerides enzymatically hydrolyzed to glycerol and fatty acids, and the resulting glycerol measured via an enzymatic reaction to generate a colorimetric signal proportional to triglyceride content. *n* = 9; *, *p* ≤ 0.05; Figure S4: Quantitative gene expression analysis of primary cultures of WT and Fst KO MEF obtained from D13 embryos. *n* = 4, **, *p* ≤ 0.01; ***, *p* ≤ 0.001; Figure S5: Metabolomic analysis (UC Davis Metabolomics Center) of ornithine (A,C) and urea (B,D) levels in liver and plasma samples obtained from 6–8-week-old male WT and follistatin transgenic (Fst^Tg^) mice (ref. [5]). Ornithine and urea levels in liver and plasma samples were quantified by targeted metabolomic analysis at the UC Davis Metabolomics Center following their standard protocols, in which metabolites were extracted from tissues and biofluids, separated by liquid chromatography, and measured by mass spectrometry against authenticated standards for accurate quantification. *n* = 12, *, *p* ≤ 0.05; **, *p* ≤ 0.01.

## Abbreviations

Fst	Follistatin
ApoE	Apolipoprotein E
LDLR	Low-density lipoprotein
AAV1	Adeno-associated virus 1
UCP1	Uncoupling protein 1
VCAM1	Vascular cell adhesion molecule 1
Arg1	Arginase 1
FGF21	Fibroblast growth factor 21
AdipoQ	Adiponectin;
MEF	Mouse embryonic fibroblast;
WAT	White adipose tissue;
PGC1α	PPAR gamma coactivator-1α
