# Supplementary material for: Follistatin Mitigates Atherosclerosis Through Activation of Arginine Metabolism and Adipose Browning
Source: Cells. 2026 Jul 2;15(13):1205. doi: 10.3390/cells15131205 (PMC13360072; doi:10.3390/cells15131205)
Supplement: Supplementary file 1 [file cells-15-01205-s001.zip › cells-4328335-supplementary.pdf]

Figure S1

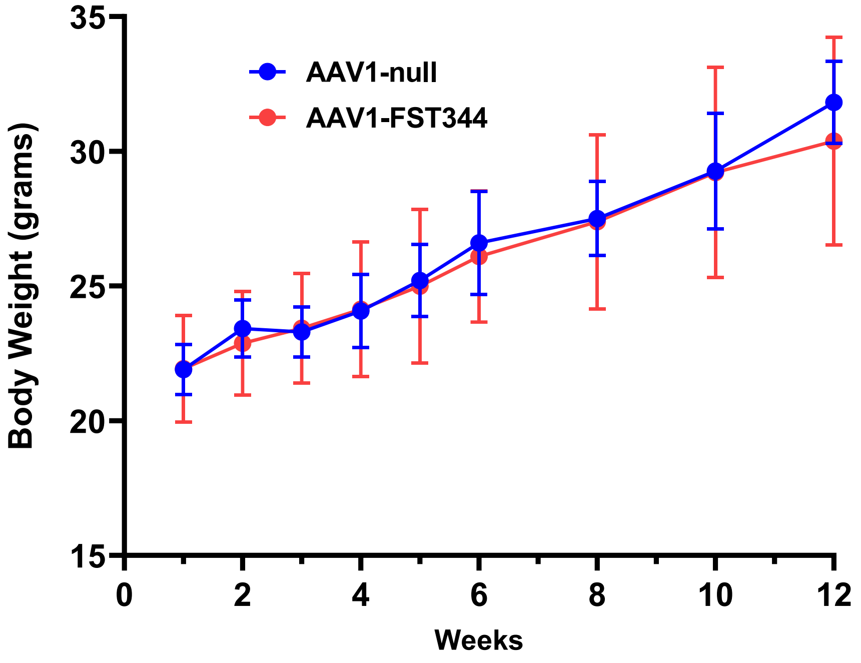

Figure S1. Weekly body weight analysis. 8-week-old *Ldlr*$^{-/-}$ male mice were injected via tail vein with 5 × 10^{11} viral particles of either AAV1-null or AAV1-FST344 or AAV1-null and fed a WD for 12 weeks. n=8-9.

Figure S2

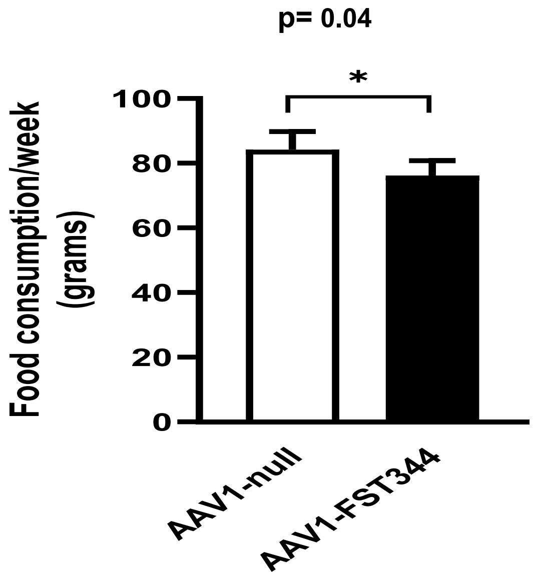

Figure S2. Analysis of weekly food consumption. 8-week old *Ldlr*$^{-/-}$ male mice were injected via tail vein with 5 × 10^{11} viral particles of either AAV1-null or AAV1-FST344 or AAV1-null and fed a WD for 12 weeks. n=8-9; *, p=0.04.

Figure S3

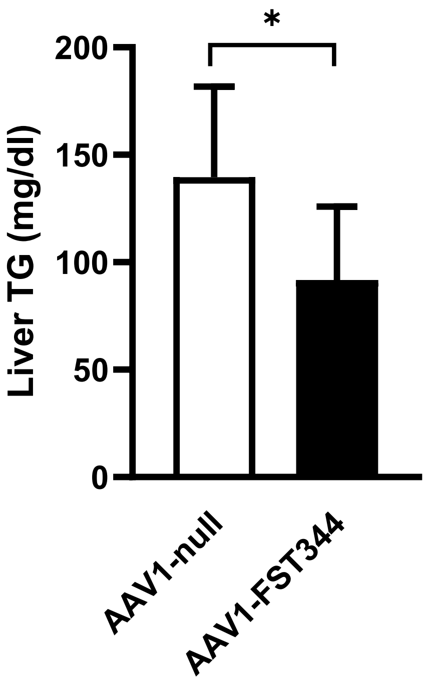

Figure S3. Analysis of liver TG levels in *Ldlr*$^{-/-}$ male mice. Mouse liver triglycerides were quantified using a commercial colorimetric assay kit (Abcam, ab65336) according to the manufacturer’s instructions, in which liver tissue was homogenized, triglycerides enzymatically hydrolyzed to glycerol and fatty acids, and the resulting glycerol measured via an enzymatic reaction to generate a colorimetric signal proportional to triglyceride content. n=9; *, $p \leq 0.05$.

Figure S4

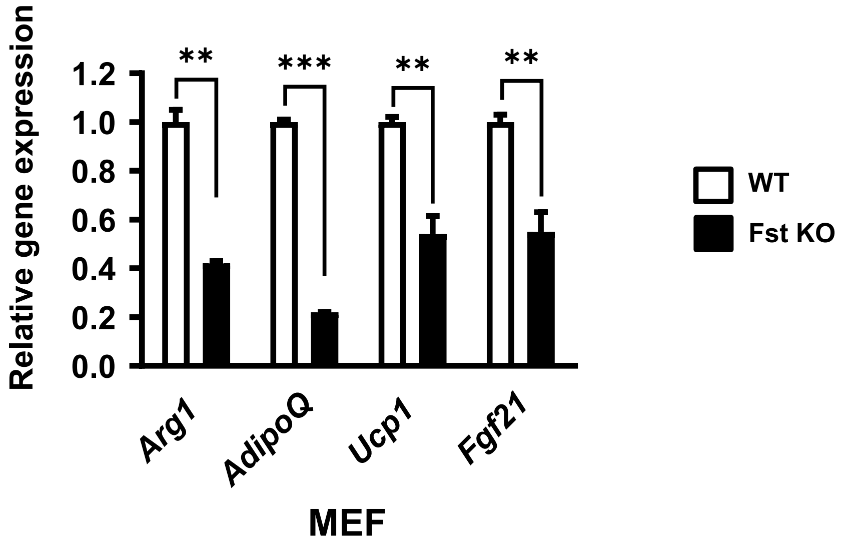

Figure S4. Quantitative gene expression analysis of primary cultures of WT and Fst KO MEF obtained from D13 embryos. n=4, **, p≤0.01; ***, p≤0.001.

Figure S5

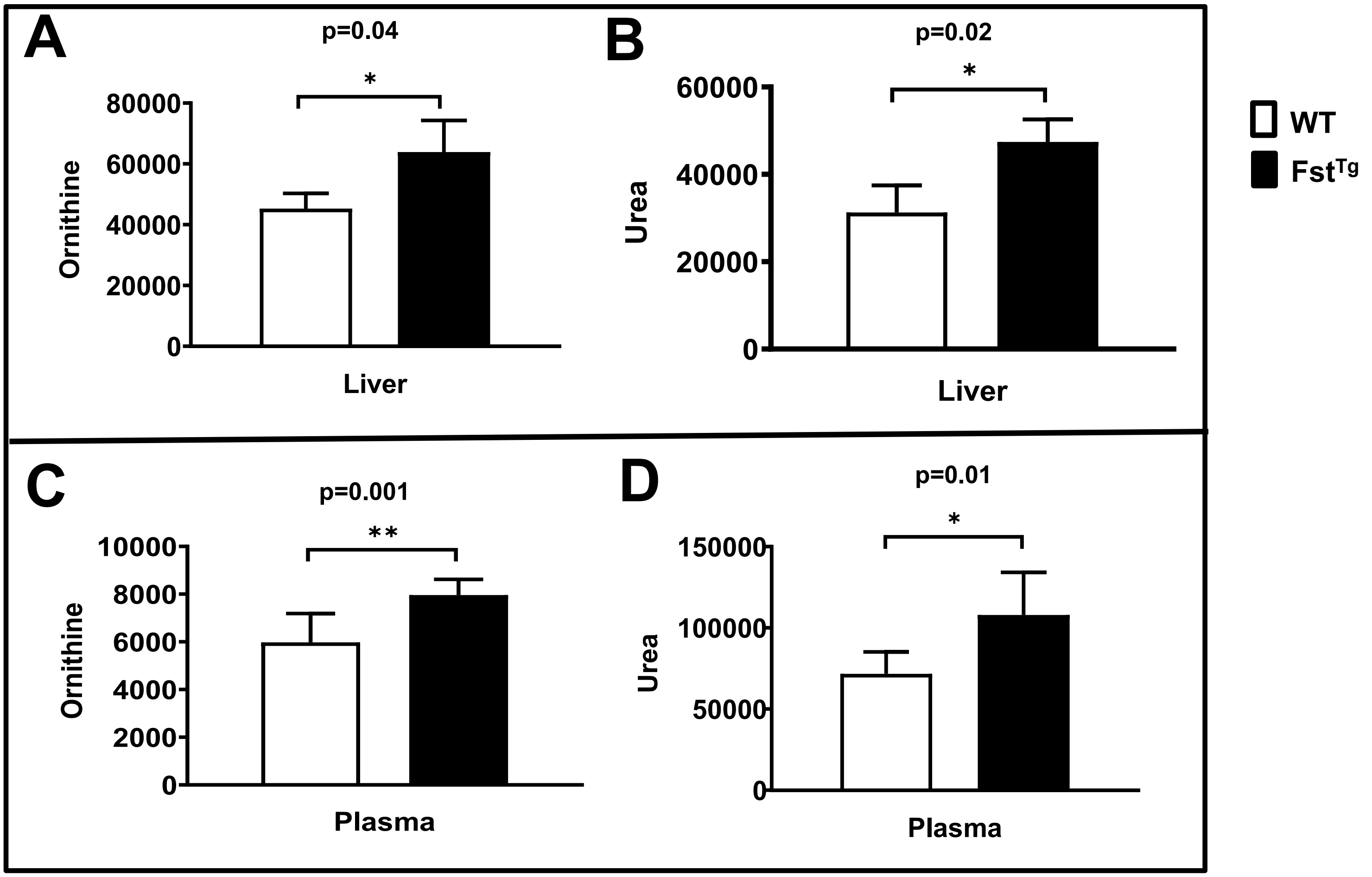

Figure S5. Metabolomic analysis (UC Davis Metabolomics Center) of ornithine (**A, C**) and urea (**B, D**) levels in liver and plasma samples obtained from 6–8-week-old male WT and follistatin transgenic (Fst^{Tg}) mice (ref 5). Ornithine and urea levels in liver and plasma samples were quantified by targeted metabolomic analysis at the UC Davis Metabolomics Center following their standard protocols, in which metabolites were extracted from tissues and biofluids, separated by liquid chromatography, and measured by mass spectrometry against authenticated standards for accurate quantification. n=12, *, $p \le 0.05$; **, $p \le 0.01$.
